# SARS-CoV-2 infection associated with micturition syncope: Our experience with 4 case reports

**DOI:** 10.1097/MD.0000000000021512

**Published:** 2020-07-31

**Authors:** Victoria Birlutiu, Rares Mircea Birlutiu, Alin Iulian Feiereisz

**Affiliations:** a Lucian Blaga University of Sibiu, Faculty of Medicine Sibiu, Academic Emergency Hospital Sibiu - Infectious Diseases Clinic, Sibiu; b Faculty of Medicine Sibiu, FOISOR Clinical Hospital of Orthopedics, Traumatology, and Osteoarticular TB Bucharest; c Academic Emergency Hospital Sibiu – Internal Medicine Clinic, Sibiu, Romania.

**Keywords:** case report, COVID-19, micturition syncope, SARS-CoV-2, syncope

## Abstract

**Rationale:**

Syncope is described as the loss of consciousness and postural muscle tone with a short duration and fast onset. Micturition syncope can be caused by abnormal vasovagal response or by the dysfunction of the blood pressure regulating mechanism, which occurs before, during, or immediately after urination.

**Patient concerns:**

We present 4 cases of COVID-19 hospitalized in the Department of Infectious Diseases of the Academic Emergency Hospital Sibiu, Romania, cases that presented micturition syncope.

**Diagnoses:**

During hospitalization, patients confirmed with SARS-Cov-2 infection using real time reverse transcriptase-polymerase chain reaction (RT-PCR) assay, presented micturition syncope in different stages of the infection (at the beginning and in the second week of evolution).

**Interventions:**

Other causes of syncope such as adrenal insufficiency secondary to corticosteroids treatment, cardiac rhythm disorders, neurological impairment, dehydration, vasoactive medication, malignancies, pulmonary hypertension and coughing were excluded. The treatment of SARS-CoV-2 infection was performed following the local and national guidelines.

**Outcomes:**

The clinical course of all 4 patients diagnosed with COVID-19 and micturition syncope was favorable. To our knowledge, micturition syncope in COVID-19 patients has yet not been reported by other authors.

**Lessons:**

To our knowledge, micturition syncope associated with the evolution of COVID-19, has yet not been reported by other authors.

## 1 Introduction

Syncope is defined as a short-term loss of consciousness with a fast onset and the impossibility of maintaining postural muscle tone. Micturition syncope can be caused by the vasovagal reflex or with the dysfunction of the blood pressure regulating mechanism (as in hypotension), which occurs before, during, or immediately after urination. It has been reported especially in male patients, associated with orthostatism, in healthy people, especially in the age group of 40 to 50 years, or in those with neurological disorders,^[1,2]^ diabetes mellitus, spinal cord injuries,^[3]^ patients that are on vasodilator medication or depression medication (selective serotonin reuptake inhibitors and tricyclic antidepressants), antipsychotics and levodopa.

As described in the literature, neoplastic diseases such as a paraganglioma of the urinary bladder^[4]^ or intermittent urinary bladder catheterization in patients with neurological disorders can be associated with micturition syncope.^[3]^ The occurrence of micturition syncope in the clinical course of SARS-CoV-2 infection in the 4 patients of different ages, with or without comorbidities, raises the issue of the association of micturition syncope with coronavirus infection.

We present 4 cases of COVID-19, hospitalized in the Department of Infectious Diseases of the Academic Emergency Hospital Sibiu, Romania with laboratory confirmation of SARS-Cov-2 infection with a positive result of real-time reverse transcriptase–polymerase chain reaction (RT-PCR) assay from nasal and pharyngeal swabs, that associated during hospitalization period micturition syncope.

## 2 Case reports

We present 4 cases of SARS-CoV-2 infection, hospitalized during April 1, 2020 until May 15, 2020, that associated micturition syncope during the disease course. Details regarding the demographic characteristics of the 4 patients, their comorbidities, and their main clinical characteristics are shown in Table 1.

**Table 1:**
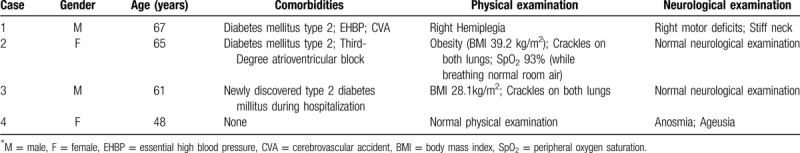
Demographic and clinical characteristics of patients.

The 1st case is the case of a 67 year old Caucasian male, known with essential high blood pressure, cerebrovascular accident at the age of 60 years, type 2 diabetes mellitus under treatment with oral antihyperglycemic agents, hospitalized for cough, retrosternal chest pain, and under treatment for the SARS-CoV-2 infection confirmed by RT-PCR assay from nasal and pharyngeal swabs. The patient was in direct contact with his son, who was also diagnosed with COVID-19 and was admitted into the hospital 10 days prior to his father‘s admission. At the time of admission on physical examination the following changes were noticed: peripheral oxygen saturation 94%, pulmonary examination without crackles on auscultation, heart rate of 76 beats per minute, blood pressure 135 over 94 mm Hg, right motor deficit predominantly at the upper limb. Chest radiography confirmed the presence of several right basilar pulmonary opacities. The most important laboratory studies are presented in Table 3. Treatment was initiated with azithromycin 500 mg in the first day, and then 250 mg/day for 4 days, associated with hydroxychloroquine, 200 mg q12h for 5 days, lopinavir/ritonavir 200/50 mg 2 tablets q12h for 10 days, insulin glargine 8 units same time every day at 22:00 (as a replacement for the oral antihyperglycemic agents), and chronic therapy with beta-adrenergic blocking agents - nebivolol 5 mg every day. On the eighth day of hospitalization, after morning micturition, the patient presented a temporary suspension of consciousness, that was repeated after he lifted himself into orthostatic position. The syncope was associated with an intense and persistent headache. Post-syncope blood pressure or blood glucose serum levels did not explain the syncopal episode. Because the patient suffered an acute traumatic brain injury, a cranial computerized tomography (CT) scan and a rapid-sequence cranial magnetic resonance imaging (MRI) were performed and revealed no pathological changes. A lumbar puncture was also performed. The cerebrospinal fluid examination was cytologically and biochemically within normal range. Mannitol, Ringers solution and B group vitamins were also included in his treatment. The patient was finally discharged on the 17th day of hospitalization when viral clearance was achieved (repeated negative RT-PCR swab test for SARS-CoV-2 at 24 hours).

**Table 3:**
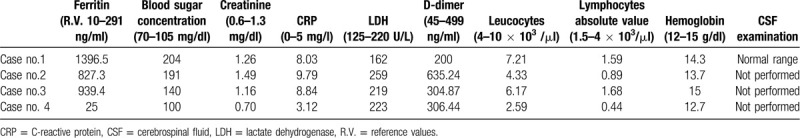
Laboratory studies.

The 2nd case is the case of a 65 year old Caucasian female, known with type 2 diabetes mellitus under treatment with insulin, essential high blood pressure, third-degree atrioventricular block, cardiac pacemaker, who was hospitalized 3 days after the onset of 38°C fever, irritative cough, asthenia and loss of appetite. The patient was confirmed with SARS-CoV-2 infection by RT-PCR assay. At the time of admission on physical examination the following changes were noticed: class II obesity with an BMI of 39.2 kg/m^2^, diffuse diminished vesicular murmur due to an increased thoracic anteroposterior diameter, bilateral basilar crackles on auscultation, peripheral oxygen saturation 93%, rhythmic heart sounds, heart rate of 80 beats per minute, blood pressure 140 over 70 mm Hg, hepatomegaly, physiological micturition and without neurosensory changes. On the 6th day of hospitalization, due to the increased areas of pulmonary crackles and in the context of a peripheral oxygen saturation of 83%, a pulmonary computerized tomography (CT) scan revealed a severity score of 20 out of 25 (the CT scan revealed the following pathological changes: multiple areas of condensation in matt glass aspect disseminated bilaterally, both peripherally and centrally, of different intensities, the densest having a halo of matt glass, and those of the anterior segment of the left lung superior lobe having a tendency to confluence, also associating fine air bronchogram. The lesions involved >75% of the right superior lobe, between 50% to 75% of the right middle lobe, >75% of the right lower lobe, between 50% and 75% of the left superior lobe, and between 5% and 25% of the left lower lobe). Data from the performed laboratory studies is presented in Table 3. The disease course was favorable under high-flow oxygen mask (10 l/minute), hydroxychloroquine 200 mg q12 h for 5 days, lopinavir/ritonavir 200/50 mg 2 tablets q12h for 10 days, dalteparin sodium 5000 IU in 0.2 ml solution 2 single dose syringe/day for subcutaneous administration for 20 days (whole hospitalization period), salbutamol sulfate 100 CFC-FREE inhaler - 100 μg/dose- 2 inhalations q12h, codeine phosphate 15 mg 3 times a day, dexamethasone 8 mg 2 times a day for 5 days, pantoprazole 40 mg/day for 5 days, paracetamol 1 g as needed and insulin. On the 11th day of hospitalization the patient, without having the need of the oxygen mask, presented a temporary suspension of consciousness for a few seconds, after the first micturition, which took place at 5 o’clock in the morning. Systolic blood pressure values were 95 mm Hg, blood glucose 191 mg/dl, a cranial computerized tomography (CT) scan was also performed and acute brain lesions (cerebral edema, intracerebral hemorrhage or tumors) were excluded. Ringer's solution 500 ml was administered. The patient was discharged on the 20th day of hospitalization when viral clearance was achieved and confirmed with 2 negative RT-PCR swab tests for SARS-CoV-2.

The 3rd case is the case of a 61 year old Caucasian male, hospitalized 3 days after the onset of dysphagia and irritative cough. The patient was confirmed with COVID-19 by RT-PCR assay from nasal and pharyngeal swabs. On physical examination, at the time of admission, the following changes were noticed: BMI of 28.1 kg/m^2^, bilateral pulmonary crackles on auscultation, peripheral oxygen saturation 93% (while breathing room air), heart rate of 94 beats per minute and blood pressure 135 over 85 mm Hg. The most relevant laboratory examinations are presented in Table 3. During the 3rd night of hospitalization, after midnight, the patient woke up for an urgent micturition. When he returned to bed he presented a temporary suspension of consciousness. After he got up, he repeated the syncope and he suffered an acute traumatic brain injury after falling. The patient was subsequently supported to maintain orthostatic position by the medical staff. A cranial computerized tomography (CT) scan and a rapid-sequence cranial magnetic resonance imaging (MRI) were performed and revealed no pathological changes. During his hospitalization period the patient received treatment with Ringers solutions, insulin, hydroxychloroquine 200 mg q12h for 5 days, lopinavir/ritonavir 200/50 mg 2 tablets q12h for 10 days and enoxaparin 60 mg in 0.6 ml, 2 single dose syringe/day for subcutaneous administration. The patient was discharged on day 15 after 2 negative tests for SARS-CoV-2 and being referred to the diabetes outpatient clinic.

The 4th case is the case of a 48 year old Caucasian female who was hospitalized 2 days after the onset of anosmia and ageusia. The patient was confirmed with SARS-CoV-2 infection by RT-PCR from nasal swabs. Physical examination was with all normal findings at the time of admission, peripheral oxygen saturation 99%, heart rate of 80 beats per minute and blood pressure 110 over 70 mm Hg. The most important laboratory studies are presented in Table 3. The next day of hospitalization, after the first morning micturition, the patient returned to the ward presenting intense headaches, diffuse abdominal pain and nausea, which precede the temporary (short duration - seconds) suspension of consciousness. Post-syncopal systolic blood pressure values were 80 mm Hg, 500 ml of saline solution being infused. A cranial computerized tomography (CT) scan was performed and acute brain lesions (cerebral edema, intracerebral hemorrhage or tumors) were excluded. During the hospitalization period the patient received treatment with hydroxychloroquine 200 mg q12h for 5 days, lopinavir/ritonavir 200/50 mg 2 tablets q12h for 10 days and enoxaparin 40 mg in 0.4 ml, 2 single dose syringe/day for subcutaneous administration. The patient was discharged on day 14 of hospitalization after 2 negative tests for SARS-CoV-2.

The first patient was under treatment with beta-adrenergic blocking agents, which can be a favoring factor for a syncope. The other 3 patients were not under treatment with hypotensive drugs or antiarrhythmic medication, diuretics or depression medication (details are shown in Table 2). Two patients repeated the micturition syncope over the next 2 minutes and suffered an acute traumatic brain injury. Among the warning signs were headaches (in 2 cases), abdominal pain and nausea (in 1 case) - details are shown in Table 2. The first 2 cases presented the micturition syncope in the second week of hospitalization and the other 2 on the first day after admission into the hospital.

**Table 2:**
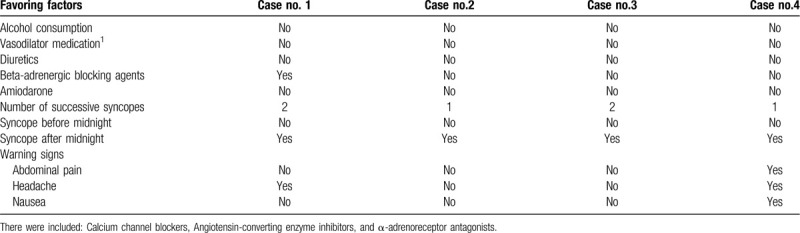
Favoring factors and warning signs.

Laboratory data revealed hyperferritinemia (in 3 cases) and elevated blood sugar concentration. The most important laboratory studies are presented in Table 3.

The cardiologic investigation of all 4 patients by a 12-lead electrocardiogram (ECG), excluded any possible paroxysmal disorders of cardiac rhythm. Post-syncope blood pressure monitoring did not highlight hypotension in any of the patients. Also, a transthoracic echocardiography was performed, which revealed no pathological changes.

We excluded other causes of syncope such as adrenal insufficiency after the use of corticosteroids, disorders of the cardiac rhythm (bradycardia, atrioventricular block, paroxysmal tachycardia and paroxysmal atrial fibrillation), neurological impairment (stroke, Parkinson's disease), dehydration, vasoactive medication, malignancies, pulmonary hypertension, and coughing.

## 3 Discussions

At the time of the writing of this manuscript, SARS-CoV-2 was responsible for 4.856.144 infected patients worldwide resulting in 318.374 deaths.^[5]^ In addition to the involvement in the severe acute respiratory syndrome, the neurotropic properties of SARS-CoV-2 are increasingly identified. Being responsible from common complaints (headache, paresthesia or disturbances of consciousness-drowsiness, irascibility) to severe neurological symptoms - ataxia, epilepsy, ischemic stroke,^[6]^ encephalitis, anosmia, ageusia,^[7–9]^ viral induced demyelination,^[10]^ the latter, most likely being caused by an immune mechanism. A multitude of psychiatric manifestations also draw attention to SARS-CoV-2, such as psychosis, anxiety,^[10]^ depression, persistent neurocognitive impairment at 18 months after discharge as in SARS patients. Manifestations that require prospective neuropsychiatric and long-term neuroimmune status follow-up ^[11,12]^ during the extended period of recovery of convalescent patients. Recently, syncope has been described as the only onset of SARS-CoV-2 infection.^[13,14]^

Angiotensin-converting enzyme 2 is involved in regulating blood pressure,^[14]^ it reduces the sympathetic nervous system activity and increases the reflex function of the arterial baroreceptors. By binding to ACE-2 receptors at an endothelial level, SARS-CoV-2 can penetrate the blood-brain barrier and cause hypoxia secondary to severe lung damage mediated by the immune response. The neurological manifestations of the patient are associated with high serum levels of the C-reactive protein and with the neutrophils count, a decreasing number of lymphocytes being a severe prognosis marker in the evolution of encephalitis in children associated with coronavirus infections.^[15]^

The micturition reflex imposes the correlation of the visceral and motor sensors with the sympathetic and parasympathetic nervous system, correlation to which is added the central voluntary control. Any of these factors, especially the imbalance between the sympathetic and parasympathetic activity, with the predominance of parasympathetic activity, contribute to the occurrence of micturition syncope. We do not exclude a possible vasodilatory action during the systemic and local cytokine storm (Interleukin-1, and Interleukin-6 promotes inflammation of the bladder mucosa) responsible for post-micturition hypotension and syncope. The significant reduction of lymphocytes was present only in 2 cases. The increases levels of D-dimer and ferritin were present in 3 out of the 4 cases.

## 4 Informed consent

Written informed consent was obtained from the patients for publication of their case report and any accompanying images. The study was accepted by the Ethics Committee of the hospital and they encouraged publishing the article. A copy of the written consent is available for review by the Editor-in-Chief of this journal.

## 5 Conclusions

We reported 4 cases of SARS-CoV-2 infection, cases that presented different clinical pictures in regarding the severity of the disease, cases which present micturition syncope in different stages of the infection (at the beginning and in the second week of evolution). Micturition syncope associated with the evolution of COVID-19, to our knowledge, has yet not been reported by other authors. Long-term prospective follow-up of COVID-19 patients is mandatory.

## Author contributions

All authors contributed equally to this manuscript in terms of acquisition, analysis and interpretation of data, conception and design, drafting the manuscript. All authors read and approved the final manuscript.

**Data curation:** Rares Mircea Birlutiu, Alin Iulian Feiereisz, Victoria Birlutiu.

**Formal analysis:** Rares Mircea Birlutiu, Alin Iulian Feiereisz, Victoria Birlutiu.

**Methodology:** Rares Mircea Birlutiu, Alin Iulian Feiereisz, Victoria Birlutiu.

**Validation:** Rares Mircea Birlutiu, Alin Iulian Feiereisz, Victoria Birlutiu.

**Visualization:** Rares Mircea Birlutiu, Alin Iulian Feiereisz, Victoria Birlutiu.

**Writing – original draft:** Rares Mircea Birlutiu, Victoria Birlutiu.

**Writing – review & editing:** Rares Mircea Birlutiu, Victoria Birlutiu.
